# Effectiveness and Coverage of Severe Acute Malnutrition Treatment with a Simplified Protocol in a Humanitarian Context in Diffa, Niger

**DOI:** 10.3390/nu15081975

**Published:** 2023-04-19

**Authors:** Pilar Charle-Cuéllar, Noemi Lopez-Ejeda, Abdoul Aziz Gado, Abdias Ogobara Dougnon, Atté Sanoussi, Nassirou Ousmane, Ramatoulaye Hamidou Lazoumar, Luis Javier Sánchez-Martínez, Fanta Toure, Antonio Vargas, Saul Guerrero

**Affiliations:** 1 Action against Hunger, C/Duque de Sevilla no. 3, 28002 Madrid, Spain; 2 EPINUT Research Group (Ref. 920325), Unit of Physical Anthropology, Department of Biodiversity, Ecology, Faculty of Biological Sciences, and Evolution, Complutense University of Madrid, 28040 Madrid, Spain; 3 Action against Hunger, Niamey BP 11491, Niger; 4 Action against Hunger, West and Central Africa Regional, Dakar BP 29621, Senegal; 5 Nutrition Direction, Ministry of Health, Niamey BP 623, Niger; 6 Centre de Recherche Médicale et Sanitaire (CERMES), Niamey BP 10887, Niger; 7 United Nations International Children’s Emergency Fund—UNICEF, 3 United Nations Plaza, New York, NY 10017, USA

**Keywords:** severe acute malnutrition (SAM), coverage, simplified approaches, ready-to-use therapeutic food (RUTF), mid-upper arm circumference (MUAC), community management of acute malnutrition (CMAM)

## Abstract

Background: the aim of this study is to evaluate the effectiveness and coverage of a simplified protocol that is implemented in health centers (HCs) and health posts (HPs) for children who are suffering from severe acute malnutrition (SAM) in the humanitarian context of Diffa. Methods: We conducted a non-randomized community-controlled trial. The control group received outpatient treatment for SAM, without medical complications, at HCs and HPs with the standard protocol of community management of acute malnutrition (CMAM). Meanwhile, with respect to the intervention group, the children with SAM received treatment at the HCs and HPs through a simplified protocol wherein the mid-upper arm circumference (MUAC) and the presence of edema were used as the admission criteria, and the children with SAM were administered doses of fixed ready-to-use therapeutic food (RUTF). Results: A total of 508 children, who were all under 5 years and had SAM, were admitted into the study. The cured proportion was 87.4% in the control group versus 96.6% in the intervention group (*p* value = 0.001). There was no difference between the groups in the length of stay, which was 35 days, but the intervention group used a lower quantity of RUTF—70 sachets versus 90 sachets, per child cured. Coverage increases were observed in both groups. Discussion: the simplified protocol used at the HCs and HPs did not result in worse recovery and resulted in fewer discharge errors compared to the standard protocol.

## 1. Introduction

Niger is one of the countries in the Sahel region, which is recurrently confronted each year with food insecurity for a large majority of its population. In the context of complex humanitarian emergencies and challenges to the socioeconomic conditions of the population, hundreds of families are forced to move due to security problems and armed conflicts. This, coupled with periods of recurrent drought and rain, raises the rate of acute malnutrition in children under 5 years of age to a critical situation [1]. One of the most seriously affected areas in the country is the Diffa region, where, based on the 2021 national retrospective mortality and nutrition survey, the prevalence of global acute malnutrition (GAM) is 16.1% (95% CI: 13.1–19.1); the prevalence of severe acute malnutrition (SAM) is 2.2% (95% CI: 1.2–4.0); and the prevalence of moderate acute malnutrition (MAM) is 13.8% (95% CI: 11.3–16.8) [2]. Together with GAM prevalence, where a high level is >15% according to WHO standards [3], there is a geographical and economic barrier to accessing service delivery.

According to Niger’s community management of acute malnutrition (CMAM) policy, treatment is separated into two distinct programs. The requirement for treatment of SAM is defined as a weight-for-height z-score (WHZ) of less than −3, per the World Health Organization (WHO) growth reference by sex and age, the presence of edema, or a mid-upper arm circumference (MUAC) of less than 115 mm. These children are treated with ready-to-use therapeutic food (RUTF) in accordance with the child’s weight as determined in a follow-up. Additionally, MAM treatment is defined as a WHZ of above −3 and below −2, or a MUAC between 115 and 125 mm, for which children receive a fixed dose of ready-to-use supplementary food (RUSF). Two different programs ensure the distribution of these two therapeutic inputs; UNICEF distributes RUTF for the treatment of children with SAM, and the World Food Programme (WFP) ensures the distribution of RUSF for children with MAM. As stated in the Ministry of Health’s National Community Health Strategy 2014–2025, the bottom level of the health pyramid is formed by health centers (HCs) and health posts (HPs) at the community level (which have to be located more than 5 km away to avoid overlap). In a HC, a nurse is normally responsible for preventive activities for children and pregnant women, as well as all curative actions, including the treatment of acute malnutrition. In a HP, there is a community health worker (CHW) who is responsible for carrying out health promotions and preventive actions, as well as curative actions for diarrhea, malaria, and acute respiratory infections—this is also known as the integrated community case management (iCCM) package of activities. This package includes SAM identification, but not its treatment (the children identified must be referred to the nearest HC for treatment) [4].

Over the last few years, the Government of Niger has made an effort to contribute to universal health coverage (UHC) and toward the Sustainable Development Goals (SDG), such as SDG 1, aimed at no poverty; SDG 2, aimed at zero hunger; and SDG 3, aimed good health and wellbeing. For these goals, the government needs to improve the case management of acute malnutrition [5,6]. Currently, the system faces major challenges, such as overburdened nurses at HCs who have to care for a high number of children per day; the difficulty and time needed to implement two different protocols for children with SAM and MAM with two different therapeutic products, which often leads to stockouts, especially regarding RUSF; as well as the geographical access barrier to health care, since 58.5% of the population lives more than a 1 h walk from the HC [7]. Family economic resources further aggravate this situation. Nearly 69% of the people in internally displaced households said they could not afford the cost of treatment, and 52% of returned households identified an inability to pay for consultation [8]. This situation is even more critical in the emergency context in Diffa, where there is also a higher safety risk to women and caregivers in traveling to bring their children to centers or posts to receive treatment over the course of several consecutive weeks.

Simplified approaches for acute malnutrition treatment are defined by UNICEF as a set of initiatives aimed at improving effectiveness and coverage, while maintaining the quality of care, as well as at reducing costs to families and health systems. Among these simplified approaches are decentralization through CHW-led treatment, and the use of a combined/simplified protocol [9]. The ComPAS (combined protocol for acute malnutrition study) is one of these simplified approaches that uses a MUAC of <125 mm as the single diagnostic criterion for acute malnutrition and involves a single therapeutic product at a fixed dose of 1000 kcal RUTF/day (two sachets a day) for children with a MUAC of <115 mm and/or + or ++ edema The protocol also includes a treatment of 500 kcal RUTF/day (one sachet a day) for children who have no edema and have a MUAC of between 115 and 125 mm. In this protocol, the amount of RUTF for children with SAM is lower than the 175 Kcal/kg/d that children receive under the standard national protocol. In South Sudan and Kenya, children receiving treatment with the ComPAS have a cured proportion that is no lower than that of children who are receiving treatment under the standard protocol [10]. A recent observational study, carried out in Mali, using the ComPAS on a cohort of 24,500 children (9710 SAM) obtained a cured proportion of 86.9% in children with SAM [11]. Based on these results, there is an international need to know the efficacy of this protocol in other contexts, especially in emergencies settings, as well as to improve the analysis of the cost-effectiveness of its implementation.

The aim of this study is to evaluate the effectiveness and coverage of the simplified ComPAS when it is applied to treat SAM at HCs and HPs in a humanitarian context in the Diffa region of Niger.

## 2. Materials and Methods

### 2.1. Study Design and Location

We conducted a non-randomized community-controlled trial involving children aged 6–59 months, who were admitted for the treatment of SAM without medical complications from December 2020 to April 2021, and who were in two health areas in the Diffa region in southeastern Niger. Children included in the study corresponded to cases that the study supervisors could transcribe from the registration sheet during their visits to each treatment site. Those children identified as having SAM with complications during the study were referred for inpatient treatment to the hospital in N’Guigmi.

The control group involved children treated in the health area of Kablewa in its HC and three HPs located in the Kawa, Kortininga, and Oudi Peulh villages. In this group, the standard CMAM protocol of the country was applied. The intervention group consisted of children treated in the N’Guigmi health area in its HC and the three HPs located in the Bonégral, Birziweya, and N’Gagala villages. Table 1. Treatment characteristics of the two study groups. In the intervention group, the ComPAS was applied with a small modification for lower-weighted children [10]. In the original ComPAS, children are admitted only by considering edema or a MUAC of <115 mm; further, they all receive a fixed dose of 2 sachets/day. In the present study, it was decided that the treatment dose for cases with a weight of less than 5 kg at admission would be reduced. According to the standard protocol, these cases would receive between 500–700 Kcal/day (the equivalent of 1.5 sachets/day). Applying the ComPAS in this way, we would have provided these children with 1000 Kcal/day (2 sachets/day), which would have markedly exceed the amount established in the Niger standard protocol. Therefore, in the present study, the children in the intervention group who were <5 kg received a reduced dose of 500 Kcal/day (1 sachet/day). In all cases, a child in the intervention group was discharged due to being considered cured when edema was resolved and when the MUAC reached ≥125 mm for two consecutive weeks.

In both groups, other treatment outcomes were recorded: death; default, when the child was absent for two consecutive follow-up visits; non-response, when weight loss was registered or anthropometric recovery stagnated in two successive visits; and referral, when medical complications appeared that required inpatient treatment. To these criteria that are outlined in the national protocol, we added another criterion of discharge error, which was for cases classified as cured in the record sheet but whose anthropometry did not meet the requirements to be classified as cured, as described for each study group. The children were rechecked once a week until they reached one of the program’s exit criteria, i.e., a MUAC of ≥125 mm and/or a WHZ of ≥−2. According to the policy in the country, children could receive treatment for a maximum of 8 consecutive weeks. They also received amoxicillin (50–100 mg/kg/day, divided twice a day for five days) and one single dose of 500 mg of mebendazole at the first visit for deworming. Both intervention sites’ groups received monthly supervision by the Action Against Hunger team. Twice during the project, the technical committee formed by the National Directorate of Nutrition, who are responsible for the Ministry of Health at the regional level, and Action Against Hunger conducted the evaluation and monitoring of the study in the field.

### 2.2. Socioeconomic Assessment

To determine the comparability of the groups and to be able to interpret the results better, a socioeconomic questionnaire was administered to a subsample of caregivers who coincidentally brought the children in for treatment on the same day the supervisor went to collect data (117 in the control group and 251 in the intervention group). This questionnaire collected the demographics, livelihood, and food security variables similar to those gathered in the Demographic Health Surveys (DHS). Dietary diversity was also assessed according to the food consumption score developed by the WFP, which is based on the frequency of consumption of 9 food groups [12]. Finally, we asked about the main barriers to treatment access, as Rogers et al. described in a study on treatment coverage which was conducted in 21 low- and middle-income countries [13].

### 2.3. Treatment Coverage Assessment

The coverage of the SAM treatment was assessed at the baseline (November 2020) and endline (August 2021) in both study areas. The standardized methodology, SLEAC (simplified lot quality assurance sampling evaluation of access and coverage), which was specifically designed for the community-based management of SAM in low-resource settings, was used [14]. This is an exhaustive cross-sectional survey, with quantitative and qualitative data collection. Further, this methodology also applies a two-stage sample. The selection of villages or clusters was achieved through systematic random selection, proportional to the size of the villages. Villages were considered the smallest administrative unit of clusters. The study was conducted as follows: (1) a sample of villages in the survey area was selected via a pre-selected sample size and a spatial sampling method; (2) a large-scale survey was conducted to find the number of current SAM cases in the program (cases covered), the number of current SAM cases not in the program (cases not covered), and the number of recovering SAM cases in the program.

### 2.4. Data Collection and Analyses

All data were disaggregated by patient and were collected digitally using the Kobo Toolkit software. A database cleaning process was performed, applying a plausibility criterion to the quantitative variables of ±4 standard deviations from the sample median to remove outliers considered potential transcription errors. Accordingly, 1 MUAC data point, 9 WHZ data points, and 17 length-of-stay (LoS) data points were eliminated. The statistical analysis was performed with the SPSS v.29 software. Qualitative variables were analyzed as percentages and compared between groups using the Chi-square test with the Yates correction or continuity correction when the expected values were less than 5 in more than 20% of the cells of the contingency tables. Since no a priori sample calculation was performed, the post hoc statistical power of the results was calculated for the primary outcome (recovery) using the GPower software [15] by applying the formula for inequality assessment of two independent proportions (Fisher’s exact test) considering two tails, an alpha error probability of 0.05 and the sample sizes finally achieved in each study group.

The normal distribution of quantitative variables was assessed through the Kolmogorov–Smirnov test. Subsequently, the means or medians were compared by applying Student’s *t*-test for the variables that showed a normal distribution and the Mann–Whitney U test for the non-normal variables. For treatment outcomes showing significant differences between the groups, a Cox regression was applied to obtain the crude and adjusted hazard ratios (HR) by including, as covariates, variables differing between the groups at admission. For treatment coverage, the Cochran–Mantel–Haenszel test was applied to assess the comparison regarding the final coverage between the study groups, adjusting for initial coverage. A bilateral hypothesis was considered in all the analyses performed with a confidence limit of 95% (*p* < 0.05).

### 2.5. Ethical Considerations

All the parents or guardians of the children included in the study signed an informed consent form to participate. The study was approved by the National Health Research Ethics Committee of Niger with the reference number 013/2020/CNERS.

## 3. Results

**Figure 1:**
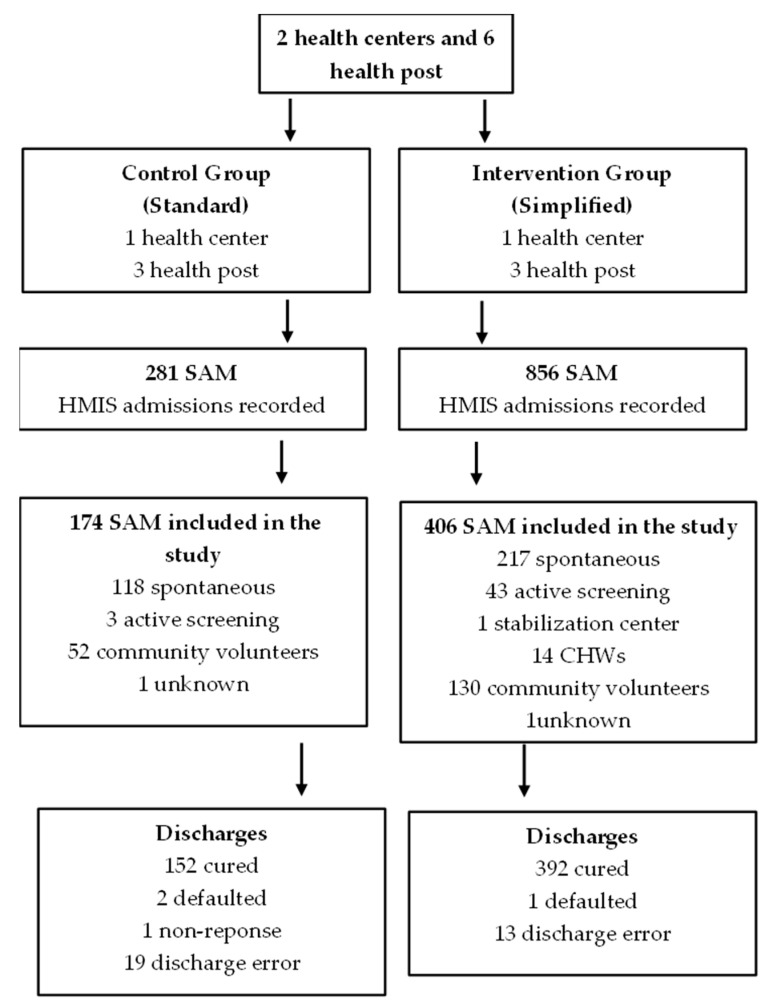
Flow diagram of cases. CHWs: community health workers; HMIS: health management information system; SAM: severe acute malnutrition.

The final sample of the study included 508 children under 5 years of age. The study sample consisted of 44.67% of the total number of children treated during the period (in accordance with the national health information system), since data collection was limited due to safety reasons. The data collection staff were instructed to transcribe cases in order of appearance in the treatment site logbook to the maximum extent possible within the time allotted at each center to avoid selection bias. Figure 1 outlines the flow of admissions to treatment outcomes within both the intervention and control groups. A total of 174 children with SAM in the control group were all treated by nurses in both HCs and HPs. A total of 406 children with SAM were treated in the intervention group, of which 256 were treated by CHWs (63.1%) and 150 were treated by nurses (36.9%) at HCs and HPs. The study groups did not differ in their average age (12.7 ± 4.9 months for the control group vs. 12.72 ± 6.0 months for the intervention group; *p =* 0.403), in the proportion of children under 12 months (112 cases = 64.4% in the control group vs. 242 cases = 59.6% in the intervention group; *p =* 0.281), or in their sex ratio (93 girls = 53.4% in the control group vs. 217 girls = 53.4% in the intervention group; *p =* 1.000).

The results of the socioeconomic survey performed during the intervention are shown in Table 2. No significant differences were found in the demographic indicators. Regarding livelihoods, the intervention group had a higher proportion of households paying for housing and a lower availability of arable land, although they also had better access to safe water and electricity. The intervention group showed greater food insecurity, with a higher proportion of families who had experienced food shortages in the last 4 weeks, albeit without significant differences in dietary diversity. In general, most families preferred to go to a health personnel when their children were sick, and there were no differences between the groups in terms of access to treatment. However, only a quarter of the samples reported their source of treatment as being more than two hours away from their homes.

**Figure 2:**
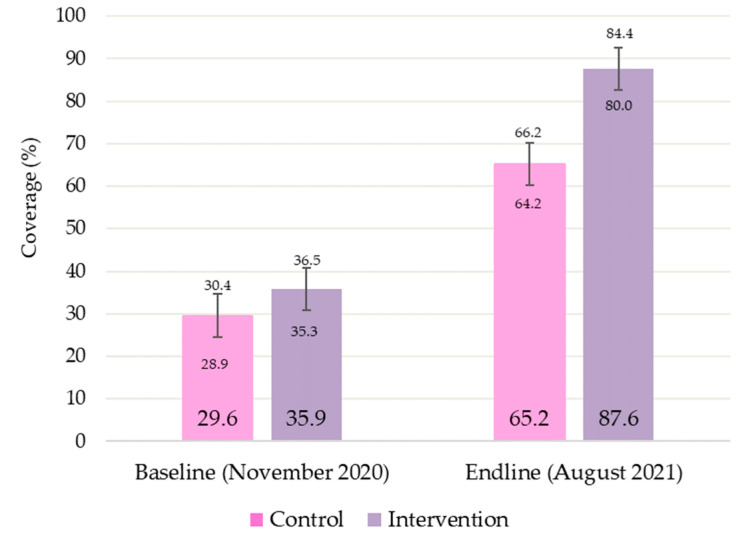
Baseline and endline coverage assessment of non-complicated severe acute malnutrition treatment compared by study groups.

Figure 2 shows the evolution of treatment coverage throughout the study in each group. In both groups, the proportion of children with SAM covered by therapeutic programs increased significantly between the baseline and endline survey (control: +35.6% *p =* 0.026; intervention: +51.7% *p* < 0.001). However, the final coverage did not differ significantly between the study groups after adjusting for initial coverage (*p =* 0.238).

Table 3 shows the characteristics of the cases at the time of admission, compared by group. Most children came to the treatment site spontaneously or were referred by community volunteers. The intervention group had fewer cases with up-to-date vaccination and more cases with comorbidities, especially diarrhea, vomiting, and coughing. No cases of edema were recorded in either study group. Regarding anthropometry at admission, the control group registered a greater severity of it by the WHZ, but no differences were found in regard to the MUAC.

The intervention group recorded a higher proportion of cured cases with a post hoc calculated power of 0.971 and an alpha error of 0.039. In addition, fewer cases were discharged erroneously without having met the cured criteria (Table 4). During treatment, 20.8% of children missed one or more non-consecutive visits with no differences between the study groups (18.8%, *N =* 30 for the control group vs. 21.7%, *N =* 75 for the intervention group; *p =* 0.441). Within the control group, all the health providers were nurses, at both HCs and HPs. Within the intervention group, there was no difference in the effectiveness of the treatment, comparing providers, nurses, and CHW. The proportion of cured cases was 94.7% (142) for cases treated by nurses and 97.7% (250) cases for cases treated by CHWs (*p =* 0.111); only one defaulted case was registered in the nurses’ group (0.7% vs. 0%; *p =* 0.787), and the proportion of discharge errors was twice as high among the nurses (4.7%; *N =* 7) compared to that among the CHWs (2.3%; *N =* 6), although the difference did not reach statistical significance (*p =* 0.322).

Table 5 shows that the mean time to recovery was 35 days in both study groups, although the children in the intervention group consumed 25 sachets of therapeutic food less, on average. The daily weight gain of the control group was significantly higher, which is consistent with the fact that they showed greater severity in their WHZ at admission. Conversely, the intervention group registered the highest daily MUAC gain.

This study included a total of 52 children weighing less than 5 kg, of whom 27 belonged to the intervention group which received only one sachet of RUTF/day, instead of the two sachets administered to children with SAM over 5 kg. In this subsample, the results showed no significant differences in the cured proportion, with 92.9% (*N =* 23) in the control group and 88.9% (*N =* 24) in the intervention group (*p =* 1.000). No deaths or referrals were registered in either group. No differences were found in the LoS (38.0 (35.0–56.0) days for the control group vs. 42.0 (35.0–50.5) days for the intervention group; *p =* 0.693). In addition, the consumption of RUTF was lower in the intervention group, although the difference was not significant (80.0 (55.0–96.0) vs. 63.0 (56.0–98.0) sachets, respectively; *p =* 0.359).

Table 6 shows the treatment results, splitting the sample according to the anthropometric indicators of acute malnutrition. In the control group, there were only 15 cases with SAM, as determined by the WHZ, and the group also had the lowest percentage of cured cases with respect to all the cases. In the children admitted only by MUAC, no differences were found in the cured proportion or the LoS. However, the RUTF sachet consumption was significantly lower in the intervention group. Of those who were admitted as having SAM by both criteria simultaneously, we found that the simplified protocol was more effective with 11% more cured cases and a lower consumption of RUTF, i.e., 24 sachets less on average.

Similarly, selecting only the most severe cases with anthropometric values in the first quartile (a WHZ of <−3.47 and/or MUAC of <110 mm), we found that the simplified protocol recorded more cured cases (93.2% and *N =* 124 vs. 82.6% and *N =* 57; *p =* 0.019), fewer discharge errors (6.8% and *N* = 9 vs. 15.9% and *N =* 11; *p =* 0.038) and no difference found in defaulters—with only one case in the control group (1.4%) and none in the intervention group (*p* = 0.738). In this group, the most severe group, the LoS was also shorter with the simplified protocol (35.0 (28.0–44.5) vs. 42.0 (29.0–56.0) days; *p* = 0.070), as was the total RUTF sachet consumption (70.0 (56.0–84.0) vs. 100.0 (80.0–120.0); *p* < 0.001).

## 4. Discussion

The results of the present study show that in a humanitarian setting, the treatment of SAM with a simplified protocol does not result in worse recovery and results in fewer discharge errors compared to the standard protocol. The socioeconomic survey showed that the intervention group households were worse off, with the highest proportion of families paying for housing, having less land available for cultivation, and being more exposed to food insecurity and food shortages in the weeks prior to the study. Even so, the proportion of children cured within the intervention group, respecting the Sphere standards for humanitarian interventions [16], was 96.6%, which is significantly higher than the 87.4% found in the control group. This difference was maintained between children who were admitted with the two anthropometric criteria of SAM simultaneously (a WHZ of <−3 and MUAC of <115 mm) and those with the highest severity of malnutrition (a WHZ of <−3.47 and/or MUAC of <110 mm). Comparing children in both groups admitted only with the MUAC criteria, the cured proportion of children was similar, being 96.1% of children in the control group, versus 96.4% of children in the intervention group with the simplified protocol.

Our results regarding SAM treatment performance when using a simplified protocol are consistent with previous evidence. One of the first experiences of using a simplified protocol with a reduced dose of RUTF was in an Action Against Hunger program in Myanmar in 2009, which was conducted to deal with a stockout of nutritional intrants. In this case, the protocol used on children with SAM involved reducing the dose according to the national protocol recommendation for stockout situations until the child reached a WHZ of >−3 or MUAC of >110 mm. Thereafter, all children received a fixed dose of 1 sachet of RUTF a day (500 kcal/day). James et al. evidenced that the cured proportion of children was 90.2% and the defaulted proportion of children was 2%, which reached the Sphere standard and were obtained with even lower doses than were used in our study [17]. There are two studies that have previously tested the ComPAS. In an observational cohort study in Mali on 9710 children with SAM, Kangas et al. found that the cured proportion was 86.9% [11]. Baley et al., in a cluster-randomized controlled non-inferiority trial in South Sudan and Kenya, found that there was no significant difference in the cured proportion compared to the standard protocol, i.e., the cured proportion was 76.3% versus 73.5%, respectively [18]. Kangas et al., in Burkina Faso, using another simplified protocol called MANGO (modeling an alternative nutrition protocol generalizable for outpatient)—which consisted of a dose reduction from the third treatment week onwards in children with SAM—found no significant difference in the proportion of those cured compared to the standard protocol, i.e., the proportion of those cured was 52.7% in the intervention group versus 55.4% in the control [19]. Despite testing the use of a simplified protocol, the effectiveness of this study could not be compared with other simplified protocols, such as OptiMA (optimising treatment for acute malnutrition), which was tested in Burkina Faso and the Democratic Republic of Congo where the researchers used a reduced dose according to the MUAC cut-off: <115 mm and 175 Kcal/kg/d; 115–120 mm and 150 Kcal/kg/d; 120–125 mm and 125 Kcal/kg/d [20], [21]. In the group with MUAC values of <115 mm, the dose of 175 Kcal/kg/day of RUTF does not represent a reduction according to the standard protocol in Niger, which is 170 Kcal/kg/day.

Our analysis found no differences between the study groups for the LoS, although the children in the intervention group had a lower average weight gain per day, which was 7.04 g/kg/day in the control group versus 5.95 g/kg/day in the intervention group. Regarding the LoS, Ogobara et al. in the Mayahi district, Niger—with a non-randomized controlled trial comparing the effectiveness and coverage of decentralized SAM treatment, with the CHWs applying the standard CMAM protocol—evidenced a LoS of 36 days, which is similar to the 35 days in the present study [22]. Compared with other contexts that have evaluated the ComPAS, Kangas et al. in Mali, with a cohort of 9,710 children, found results that are similar to those of the present study. The overall weight gain velocity was 5.8 g/kg/d among children with an MUAC of <115, 5.95 g/kg/d in our study. The present study, however, obtained better results in terms of the LoS of 35 days compared with the LoS of 54.5 days in the research conducted in Mali [11]. In the study conducted in South Sudan and Kenya, the mean LoS was 65 days (almost twice as much as that in the present study) and the weight gain was 1.9 g/day (three to four times lower than that in the present study), with no significant difference between the standard and simplified protocols being found. This was explained by the fact that in both the contexts of Sudan and Kenya, the treatment adherence was lower when compared with that in Mali and in the present study [18]. Although there was no difference in the proportion of cured children, and as our results are consistent or better than those of the previous ComPAS studies, the intervention group that received less RUTF had a lower weight gain. This could be explained by the severity at admission, with a higher proportion of children in the control group with a lower WHZ, and with the families’ and children’s conditions at admission. In the control group, a higher proportion of families reported no food shortage in the month prior to the survey than those in the intervention group did. Within the intervention group, a greater number of children presented associated illnesses, such as diarrhea or malaria in the previous week. Future research is needed to evaluate if this could have an impact on relapses during the coming months after being discharged.

In relation to RUTF consumption, the intervention group of our study consumed fewer sachets per SAM-cured child (70 vs. 95 in the standard protocol group). The lower WHZ in the control group could have contributed to this difference in the number of sachets of RUTF. The quantity of RUTF in our intervention group was lower than that in the ComPAS study conducted in Mali, where children with SAM used 96 sachets per child cured, which is consistent with the higher LoS in the study [11]. Kangas et al. also showed a reduction in sachets when using the combined protocol, reporting 105 vs. 148 sachets of RUTF per cured child in Kenya, and 133 vs. 209 sachets of RUTF per child cured in South Sudan. The lower amount used in our study could be explained by the shorter LoS, as well as the fact that the group of under 5 kg children received only one RUTF sachet per day and not two, as in the study conducted in Kenya and South Sudan [18]. It was not possible to compare the amount of RUTF used in our study with that used in the MANGO project because, in this case, there was no fixed amount of sachet per child. The RUTF was administered according to the child’s weight, which was different for each child and thus made it impossible to find out the average number of sachets per child discharged due to being considered cured [23].

In terms of coverage assessment, we have shown an increase in SAM treatment access in both groups with the standard CMAM protocol and the simplified protocol with no significant difference between both. The previous study with the ComPAS in South Sudan and Kenya did not show an effect on treatment coverage in either the control or intervention groups. This is explained by the fact that, in our study, in both groups, there was a decentralization of treatment with respect to the level of the HPs, which increased service delivery sites and brought them closer to families. This result is consistent with those of previous non-randomized controlled studies that were conducted with CHW-led treatment. In the groups where CHWs treated SAM outside health facilities, there was an increase in coverage at the end of the intervention of up to 86.7% in Mali, 68.8% in Niger, 78% in Mauritania, and 80.9% in Tanzania [22,24,25,26]. Kodish et al. assessed the factors that may impact treatment coverage for malnutrition in Niger and identified the distance to HCs and the associated transport costs as one of the three main barriers to accessing treatment. These are the barriers that are being broken by including CHWs as treatment providers [27]. Maust et al., in a cluster-randomized control trial in Sierra Leone, evaluated the relationship between the coverage and management of acute malnutrition via an integrated protocol. In that study, an integrated protocol in the HC was applied; in comparison to the standard protocol, theirs consisted of a single diagnostic criterion of a MUAC of <125 mm and a single therapeutic product with RUTF at a fixed dose for both SAM or MAM. In this study, even without the decentralization treatment being administered by CHWs, an increase in treatment coverage was shown in the group using an integrated protocol (of 71% compared to 55% in the standard protocol group). In this case, the treatment coverage was for GAM, thus including children with SAM and MAM. However, in the present study, and in those referenced above, the treatment was only related to SAM coverage [28].

Along with the performance of the protocol, from an implementation point of view, it is important to highlight the quality with which the management of acute malnutrition protocols are applied. This study analyzed errors in children who were discharged due to being considered cured, i.e., those who were discharged without having reached the WHZ and/or MUAC criteria. The intervention group with a simplified protocol had a significantly lower error proportion than the control group: 3.2% vs. 10.9%. Even within the group of children who were admitted with more severe anthropometric conditions, this difference remained significant (6.8% vs. 15.9%). This is an element, along with the effectiveness and coverage results, which is important to take into consideration, so that the simplified protocol can be used under exceptional circumstances [29]. In relation to the use of this simplified protocol that uses only the MUAC as the sole diagnostic criterion, we can mention that in the specific context of our study, only 9.1% of the children admitted were identified with WHZs as the sole diagnostic criterion. The MUAC captures most children in this setting. Similar results were obtained in another region of Niger, Mayahi, with 8.6% and 14.1% of SAM admission being diagnosed only with the WHZ in the control and intervention group (compared to 25.8% and 37.7% of SAM cases diagnosed by the MUAC only) [22].

The main limitation of this study was that it was not a randomized controlled trial, so the results cannot be extrapolated to other contexts and the probability of residual confounding is increased. Although there was no difference between the groups, the proportion of cured cases was high, so these results should be taken with caution in other contexts where the proportion of cured cases is not as high as this one. Another possible limitation is the difference in sample sizes between groups, whereby there was a higher incidence of cases in the intervention area. However, this did reflect the reality of the field, where the worst socioeconomic conditions are recorded, with a higher population of 47,198 habitants vs. a population of 26,176 habitants in the intervention and control groups, respectively. Nevertheless, the statistical tests used are robust with regard to this type of sample imbalance and the results show that the simplification of the protocol is effective even with worse baseline conditions. The coverage surveys were carried out in different time periods, just 8 months later after the start of the project. The SQUEAC methodology suggests that this kind of survey can be implemented in periods of a minimum of 4 months after the new intervention is launched for monitoring the effect [13]. To our understanding, one of the factors that may have a negative effect on treatment coverage is the overburdening of health facilities and worsening of access to treatment sites due to flooding, which means not all children in need can reach them. In our case, the period of the highest prevalence of the disease was when the final survey was conducted, and, even with this situation, an increase in treatment coverage was shown in both groups when we decentralized treatment to the community level.

## 5. Conclusions

To the best of our knowledge, this is the first study in West Africa to demonstrate the effectiveness and coverage of SAM treatment using a simplified protocol in a complex humanitarian context. The use of a MUAC of <115 mm as the sole diagnostic criterion and a RUTF dosage fixed at two sachets a day for children over 5 kg, as well as a dosage of one sachet a day for those under 5 kg, resulted in a cured proportion of 96.6%, a defaulted proportion of 0.2%, and a decrease in the amount of RUTF, with 70 sachets per cured child. These results show that there are fewer errors in the simplified protocol’s application than in the application of the standard CMAM protocol.

In the Niger context, the evidence from this study complements the results obtained in a previous study that was conducted with another simplified approach, CHW-led treatment. Both results can be considered by policy-makers for the application of a simplified approach under exceptional circumstances, as in the case of Diffa. In this context, where there is a situation of insecurity and a lack of access to health services, together with logistical problems leading to usual stockouts of the two nutritional inputs, the continuity of care of children suffering from acute malnutrition cannot be guaranteed. Therefore, the use of simplified approaches can contribute to the treatment of more children more effectively.

## Figures and Tables

**Table 1:** Treatment characteristics of the two study groups.

	Control Group (Standard)	Intervention Group (Simplified)
Treatment providers	Nurses at health facilities and health posts	Nurses and CHWs at health facilities and health post
Follow-up frequency	Weekly	Weekly
Admission criteria	Edema (+) or WHZ with a <−3 z-score or a MUAC of < 115 mm	Edema (+) or a MUAC of <115 mm
Product and dosage	RUTF/weight: 170 Kcal/kg/day	RUTF fixed dosage: 2 sachets/day (= 1000 Kcal/day) *
Recovery criteria	No edema and a WHZ of ≥1.5 or a MUAC of ≥125 mm	No edema and a MUAC of ≥125 mm

CHWs: community health workers; MUAC: middle-upper arm circumference; RUTF: ready-to-use therapeutic food; SAM: severe acute malnutrition; WHZ: weight-for-height z-score. * 1 sachet/day (=500 Kcal/day) if the weight at admission was <5 kg.

**Table 2:** Sociodemographic characteristics of cases at admission for severe acute malnutrition treatment, compared by study group.

	Indicator	Control (Standard)		Intervention (Simplified)		
Demographics		**N**	**Mean (SD)** **or % (*n*)**	**N**	**Mean (SD)** **or % (*n*)**	***p*-Value**
	Number of cohabiting people	117	5.5 (2.4)	251	6.0 (2.8)	0.110
	Number of children under 5 years of age living with the treated child	115	1.8 (1.3)	237	1.5 (1.1)	0.066
	Education of primary caregiver (years)	117	0.19 (0.49)	251	0.28 (0.68)	0.187
Livelihoods	Type of housing	117		241		
	Property		88.0% (103)		58.1% (140)	<0.001
	For rent		0.9% (1)		12.9% (31)	<0.001
	On loan		11.1% (13)		29.0 (70)	<0.001
	Households with access to safe water	117	38.5% (45)	251	55.8% (140)	0.003
	Households with access to safe sanitation	117	0.0% (0)	251	1.6% (4)	0.405
	Households with electricity	117	3.4% (4)	251	15.5% (39)	0.001
	Households with arable land	117	29.9% (35)	251	4.8% (12)	<0.001
	Households with livestock	117	48.7% (57)	251	53.4% (134)	0.470
	Number of cows	57	6.7 (9.8)	134	8.4 (13.3)	0.329
	Number of sheep	57	4.6 (8.5)	134	2.0 (4.3)	0.030
	Number of goats	57	8.7 (11.2)	134	12.2 (14.8)	0.070
	Households with a construction floor (concrete, cement, wood, tiles, etc.)	117	0.0% (0)	251	1.2% (3)	0.572
	Households with a construction roof (concrete, cement, wood, tiles, etc.)	117	0.0% (0)	251	1.2% (3)	0.572
Food security	Number of meals per day	108	2.8 (0.5)	221	2.9 (0.4)	0.819
	Lack of food in the last 4 weeks	116		247		
	Never		88.8% (103)		61.1% (151)	<0.001
	Rarely		10.3% (12)		36.0% (89)	<0.001
	3–10 times		0.9% (1)		2.4% (6)	0.546
	More than 10 times		0.0% (0)		0.4% (1)	0.999
	Food diversity	117	62.3 (21.9)	251	62.8% (20.2)	0.825
	Poor diet		7.7% (9)		2.4% (6)	0.035
	Limited diet		5.1% (6)		4.8% (12)	0.999
	Acceptable diet		87.2% (102)		92.8% (233)	0.116
Health care access	Behavior if the child is sick	116		251		
	Health post or CHW		99.1% (115)		92.8% (233)	0.022
	Traditional medicine		0.0% (0)		6.8% (17)	0.009
	Self-medication		0.9% (1)		0.4% (1)	0.999
	Households with difficulties in access	117	3.4% (4)	251	1.2% (3)	0.296
	Time needed to reach treatment	117		251		
	30 min or less		32.5% (57)		41.3% (104)	0.230
	Up to 1 h 30 min		47.0% (31)		31.6% (79)	0.396
	More than 2 h		20.5% (24)		27.1% (68)	0.219

CHW: community health worker; SD: standard deviation.

**Table 3:** Characteristics of cases at admission for severe acute malnutrition treatment, compared by study group.

	Control (Standard) N *=* 173	Intervention (Simplified) N *=* 405	*p*-Value
Referred by:	% (*n*)	% (*n*)	
Spontaneous	68.2% (118)	53.6% (217)	0.039
Active screening campaigns	1.7% (3)	10.6% (43)	
Stabilization centers	0% (0)	0.2% (1)	
CHWs	0% (0)	3.5% (14)	
Community volunteer	30.1% (52)	32.1% (130)	
Immunization up to date	55.6% (95)	45.7% (179)	0.031
Comorbidities in the last 7 days:	% (*n*)	% (*n*)	
Diarrhea	3.5% (6)	14.9% (60)	<0.001
Vomit	0% (0)	3.0% (12)	0.048
Fever	1.7% (3)	8.7% (35)	0.002
Cough	3.4% (6)	18.1% (73)	<0.001
Acute respiratory infection	0.6% (1)	1.0% (4)	0.980
Malaria-positive	0% (0)	1.2% (5)	0.327
Pale conjunctiva	1.4% (3)	4.7% (19)	0.089
Edema (mild)	0% (0)	0% (0)	--
Anthropometry			
Weight (kg); mean (SD)	6.2 (1.1)	6.3 (1.2)	<0.001
Height (cm); mean (SD)	69.1 (6.5)	70.2 (6.8)	0.068
WHZ; mean (SD)	−3.17 (1.02)	−2.67 (1.31)	<0.001
WHZ < −3.47; %(*n*) *	30.1% (52)	21.9% (88)	0.036
MUAC; mean (SD)	111.1 (5.2)	111.3 (4.0)	0.883
MUAC < 110 mm; %(*n*) *	17.2% (30)	15.0% (61)	0.501

CHWs: community health workers; MUAC: mid-upper arm circumference; SD: standard deviation; and WHZ: weight-for-height z-score. * Most severe cases applied to the quartile one value as the cut-off point for the WHZ and MUAC.

**Table 4:** Discharge outcomes compared by study group.

Treatment Outcomes	Control (Standard)	Intervention (Simplified)	ꭓ^2^ *p*-Value	Crude HR (95% CI)	* Adjusted HR (95% CI)
Cured	87.4% (152)	96.6% (392)	<0.001	1.242 (1.026–1.504)	1.300 (1.055–1.601)
Defaulted	1.1% (2)	0.2% (1)	0.449	--	--
Non-response	0.6% (1)	0% (0)	0.662	--	--
Referred	0% (0)	0% (0)	--	--	--
Death	0% (0)	0% (0)	--	--	--
Discharge error	10.9% (19)	3.2% (13)	<0.001	0.298 (0.135–0.656)	0.283 (0.112–0.713)

CI: confidence interval; HR: hazard ratio. * Adjusted by anthropometry, comorbidities, and vaccination status at admission.

**Table 5:** Treatment duration, product consumption, and the anthropometric gain of cured children, compared by group.

	Control (Standard)	Intervention (Simplified)	*p*-Value
	Median (IQR)	Median (IQR)	
Length of stay (days)	35.0 (29.0–47.0)	35.0 (28.0–42.0)	0.254
RUTF (sachets)	95.0 (74.0–120.0)	70.0 (56.0–77.0)	<0.001
Weight gain (g/kg/day)	7.04 (5.03–9.76)	5.95 (3.62–8.40)	<0.001
MUAC Gain (mm/day)	0.43 (0.31–0.50)	0.46 (0.37–0.57)	0.001

IQR: interquartile range; MUAC: mid-upper arm circumference; and RUTF: ready-to-use therapeutic food.

**Table 6:** Treatment results by anthropometric classification at admission compared by group.

		Control (Standard) N *=* 165	Intervention (Simplified) N = 379	*p*-Value
WHZ only (<−3 z-score)	Total cases, % (*n*)	9.1% (15)		
	Cured, % (*n*)	80.0% (12)		
	Length of stay(days), median (IQR)	32.0 (21.8–44.3)		
	Admission of RUTF to recovery, median (IQR)	100.0 (60.0–115.0)		
MUAC only (<115 mm)	Total cases, % (*n*)	30.9% (51)	58.6% (222)	
	Cured, % (*n*)	96.1% (49)	96.4% (214)	1.000
	Length of stay(days), median (IQR)	35.0 (29.3–42.8)	35.0 (28.0–43.0)	0.803
	Admission of RUTF to recovery, median (IQR)	95.0 (75.0–120.0)	70.0 (56.0–84.0)	<0.001
WHZ and MUAC	Total cases, % (*n*)	60.0% (99)	41.4% (157)	
	Cured, % (*n*)	85.9% (85)	96.8% (152)	0.001
	Length of stay (days), median (IQR)	38.0 (28.0–52.0)	35.0 (28.0–42.0)	0.071
	Admission of RUTF to recovery, median (IQR)	94.0 (59.0–112.5)	70.0 (56.0–72.0)	<0.001

IQR: interquartile range; MUAC: mid-upper arm circumference of <115 mm; RUTF: ready-to-use therapeutic food sachets; and WHZ: weight-for-height z-score of <−3.

## Data Availability

The data presented in this study are available upon request from the corresponding author.
